# Does the process map influence the outcome of quality improvement work? A comparison of a sequential flow diagram and a hierarchical task analysis diagram

**DOI:** 10.1186/1472-6963-10-7

**Published:** 2010-01-07

**Authors:** Lacey Colligan, Janet E Anderson, Henry WW Potts, Jonathan Berman

**Affiliations:** 1 Medical Informatics Systems Engineering Training Program (MINDSET), Systems and Information Engineering, University of Virginia, Charlottesville VA 22903, USA; 2 NIHR King's Patient Safety and Service Quality Research Centre, King's College London, Strand Bridge House, 138-142 Strand, London WC2R 1HH, UK; 3 Centre for Health Informatics and Multiprofessional Education (CHIME), UCL Medical School, University College London, Archway Campus, Highgate Hill, London N19 5LW, UK; 4 Greenstreet Berman Ltd Fulcrum House, 5 Southern Court, South Street, Reading RG1 4QS, UK

## Abstract

**Background:**

Many quality and safety improvement methods in healthcare rely on a complete and accurate map of the process. Process mapping in healthcare is often achieved using a sequential flow diagram, but there is little guidance available in the literature about the most effective type of process map to use. Moreover there is evidence that the organisation of information in an external representation affects reasoning and decision making. This exploratory study examined whether the type of process map - sequential or hierarchical - affects healthcare practitioners' judgments.

**Methods:**

A sequential and a hierarchical process map of a community-based anti coagulation clinic were produced based on data obtained from interviews, talk-throughs, attendance at a training session and examination of protocols and policies. Clinic practitioners were asked to specify the parts of the process that they judged to contain quality and safety concerns. The process maps were then shown to them in counter-balanced order and they were asked to circle on the diagrams the parts of the process where they had the greatest quality and safety concerns. A structured interview was then conducted, in which they were asked about various aspects of the diagrams.

**Results:**

Quality and safety concerns cited by practitioners differed depending on whether they were or were not looking at a process map, and whether they were looking at a sequential diagram or a hierarchical diagram. More concerns were identified using the hierarchical diagram compared with the sequential diagram and more concerns were identified in relation to clinical work than administrative work. Participants' preference for the sequential or hierarchical diagram depended on the context in which they would be using it. The difficulties of determining the boundaries for the analysis and the granularity required were highlighted.

**Conclusions:**

The results indicated that the layout of a process map does influence perceptions of quality and safety problems in a process. In quality improvement work it is important to carefully consider the type of process map to be used and to consider using more than one map to ensure that different aspects of the process are captured.

## Background

Improvement efforts in health care have generated a variety of quality improvement and risk assessment methodologies such as Lean [1,2], Six Sigma [3,2], Healthcare Failure Modes and Effects Analysis (HFMEA) [4] and PDSA cycles [5]. A common requirement of these methodologies is to understand the existing system before attempting to implement improvement strategies: "Before improvements can be identified for a process, the process's *anatomy*, or steps, must be understood" [6]. Process mapping is therefore a central component of quality improvement efforts in healthcare [7]. Anecdotal evidence suggests that most healthcare improvement efforts are based on a sequential flow diagram, but there is little discussion in the literature about which methods are available and how to choose the most appropriate mapping method. Moreover, the possible influence of the type of process map on the perception of quality and safety problems has not been investigated. This study investigated whether the form of a process map influences practitioners' judgements about which parts of the process are risky.

Research from different traditions has provided evidence that the way information is organized and displayed affects people's performance and their interaction with that information. Human factors research shows that the arrangement of information in visual displays affects people's ability to efficiently extract and process information (see for example, [8]). Many studies of the artefacts used by healthcare professionals in their work reveal how workers use artefacts to support their cognitive work and develop shared understandings of the status of their work [9-11]. A study of physician investigators' decisions about whether to continue a clinical trial found that the method of displaying the data significantly influenced the accuracy of their decisions [12]. Recently, Miller [13] found that physicians' and nurses' diagnostic reasoning was different when they used a new display of patient information that grouped information according to physiological function, compared with a traditional patient chart. Artefacts therefore shape and structure cognitive activity.

Cognitive science research has also shown the importance of external representations on performance of a variety of problem solving activities [14,15]. This research shows that external representations are not simply lists of inputs or memory aids but are integral to a task and how it is performed. External representations are directly perceived without the need for further interpretation and provide a structure for cognitive activity by constraining the range of possibilities. Crucially, the format of a representation can determine what information can be perceived and what aspects of the problem space are explored especially if a task is novel or involves aspects of discovery [15].

In quality improvement work, process maps are artefacts that assist workers to identify areas to intervene to improve safety and quality, a task that involves examining the process from a new perspective in order to discover where the greatest risks exist. Process maps are external representations of the system and become tools for problem solving, reasoning, and decision-making about risks and improvements. The type of external representation used for quality improvement work in healthcare is likely to be crucial in ensuring the effectiveness of the quality improvement work that is carried out.

In this study we examined a community-based anti-coagulation (CBAC) clinic and used this setting to compare healthcare practitioners' risk perceptions using two different types of process maps relevant to healthcare improvement: a sequential flow diagram and a hierarchical task analysis diagram. We chose these two different diagrams because they represent the two main ways of organising task information diagrammatically [16]. These two layouts for process maps have also been discussed by others considering how best to represent tasks and processes in healthcare (for example, [17]) but there is no information available about the strengths and limitations of each in this context.

Sequential flow diagrams and hierarchical task analysis diagrams (HTA) are fundamentally different. Flow diagrams present the discrete steps in a process sequentially in the order in which tasks are accomplished. The form of a sequential flow map is free so two sequential flow maps of the same process could take very different forms. HTA results in a hierarchical diagram that organizes human work by goal, not by procedural step. High level goals are achieved by carrying out a number of sub-goals, so dependencies are represented in the hierarchical structure. HTA does not implicitly specify when things need to be done, although the diagram can be annotated with instructions about the required order of tasks. HTA diagrams have a specified structure, which means that two diagrams of the same process constructed by different people should have a very similar layout. Despite being widely used in human factors and other disciplines, HTA is not used extensively in healthcare.

### Clinical setting

Anti-coagulation is an important prophylaxis for thrombosis and embolism. The main treatment used, Warfarin, has a narrow therapeutic index, which means it must be carefully monitored and adjusted during the course of the treatment, with patients typically attending for blood tests every few weeks. The international normalised ratio (INR) is the metric used for the tendency of blood to clot. Patient safety organizations have highlighted anti-coagulation safety as a critical goal. The North Central Community-Based Anti-Coagulant and Stroke Prevention services are based at the Whittington Hospital and involve multiple Primary Care Trusts (PCTs) in north London. These services have been running for several years: they involve common governance and training arrangements, and mostly share a common combined electronic patient record/decision support system and point-of-care blood testing, but there is some variation between PCTs in the precise format of anti-coagulant clinics. The clinic structure under study is that for Barnet PCT.

In this care model, hospital-based pharmacists run clinics located in community sites in the borough of Barnet. These pharmacists have received tailored training in Warfarin management and the use of the computer system, and they work under the supervision of a hospital-based consultant cardiologist. Compared to the existing hospital-based service in which the patient undergoes venepuncture and the INR result is reviewed later by a junior doctor, the community-based service is novel in its use of pharmacists to run the clinics, point-of-care testing, the combined electronic patient record/decision support system, and its community location. Point-of-care testing with a coagulometer allows the pharmacist to obtain an immediate INR result with a finger prick. The electronic patient record supports patient tracking, audit and quality functions. The pharmacist provides face-to-face education, dose adjustment and plans the interval before the next monitoring appointment.

### Aim

The overall aim of the study was to investigate whether different external representations of a healthcare process affect healthcare practitioners' perceptions of risk and therefore indirectly the quality improvement work that is undertaken. We examined the process for managing patients using anti-coagulation medications in a CBAC clinic and investigated whether practitioners identified the same safety concerns on a sequential flow map and a hierarchical task analysis diagram. We also investigated healthcare practitioners' judgments of the usefulness of a sequential flow diagram and a hierarchical task analysis for a range of different tasks. A secondary aim of the study was to examine the process of producing these representations and to draw some conclusions about the methodological issues that arise when mapping a healthcare process using sequential flow diagrams and hierarchical task analysis.

## Methods

The study protocol was reviewed and approved by the NHS Research Ethics Committee.

### Producing the external representations

We developed two representations of the CBAC service: a sequential flow diagram and a hierarchical task analysis diagram (HTA) using the methods described by Nelson et al [6] and Stanton [16] respectively. We used multiple methods to gather information about how the service operates. Two senior pharmacists and a clinic pharmacist in the CBAC service, all of whom had extensive knowledge and experience of the service and had been involved in the Barnet service since its inception, participated as subject matter experts. Two interviews were conducted with each of the senior pharmacists to gain knowledge of the processes. The clinic pharmacist provided a talk-through and explanation of the patient management process and demonstrated how the decision support system operated. One researcher attended a training session for new CBAC practitioners. Protocols, policies and other documents were also analysed.

A draft sequential flow diagram and a draft HTA diagram were produced by the research team based on the data collected. The content of the two diagrams was controlled to ensure that they each had the same number of steps and covered the same aspects of the CBAC processes. The diagrams were reviewed for accuracy by one of the senior pharmacists who participated in the initial interviews and the diagrams were amended to incorporate his feedback. In our development of the diagrams, we made reflective notes about the challenges of producing such representations and the usually implicit choices that go into them so that we could draw some conclusions about the mapping process.

**Figure 1:**
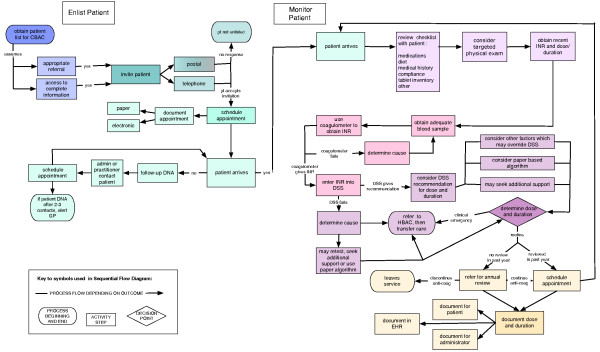
**Sequential flow diagram of the Community-Based Anti-Coagulation Clinic**.

**Figure 2:**
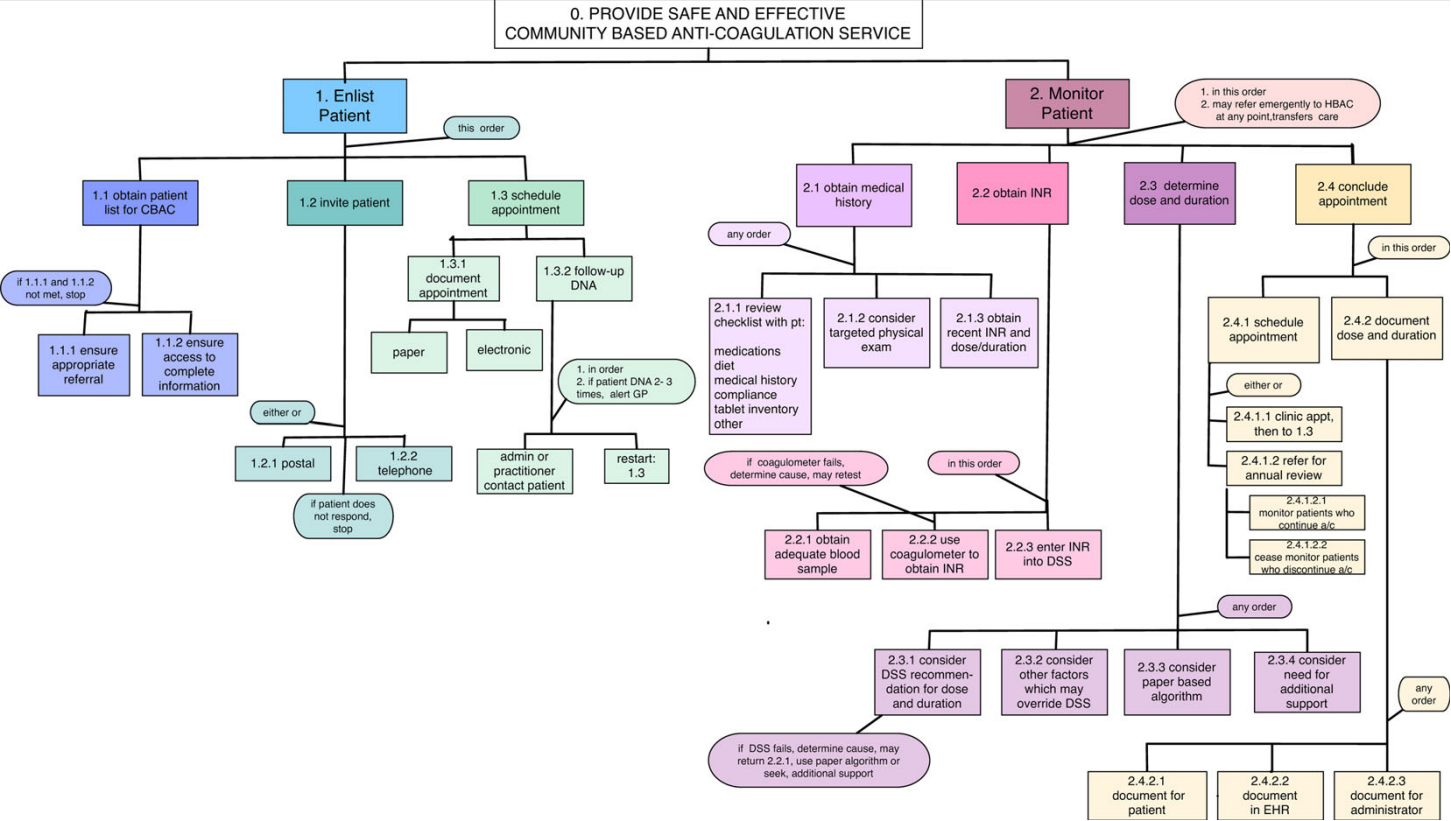
**Hierarchical task analysis diagram of the Community-Based Anti-Coagulation Clinic**.

The sequential process map is shown in Figure 1 and the HTA diagram is shown in Figure 2.

### Using and evaluating the external representations

The procedure for this phase of the study was pilot tested and refined with a group of postgraduate students working in healthcare and studying a module in patient safety.

#### Participants

The five pharmacists and two administrators who worked at the CBAC service participated in this phase of the study. None of them had participated in the previous phase of the research. Their length of employment at the CBAC ranged from four months to four years. Participants were recruited via email communication describing the study and inviting them to take part.

#### Procedure

Before taking part in the study participants received information about the study and signed consent forms. An interview was conducted with each participant. The interviews were audio taped with the participants' agreement.

There were three parts to the interview. First, without looking at the diagrams, participants were asked to identify their views of safety and quality problems in the CBAC service. Second, they were shown the diagrams individually in a counter-balanced order. They received an explanation of each diagram and were invited to review it for accuracy and then to circle on the diagram any areas of the process that were outside of their knowledge of the system and any areas where they perceived there to be safety and quality concerns. When the second diagram was presented, they were informed that the diagrams contained the same information but were not prompted to circle either the same or different concerns on the second diagram.

Third, the participants were asked to complete a short questionnaire about the usefulness of the diagrams in different communication and improvement contexts. They were asked whether they would prefer the flow diagram, the HTA, or had no preference for the following purposes:

1. If they had to explain their work to someone outside the workplace,

2. If they had to discuss a problem with a colleague,

3. If they had to explain their work to a manager in order to gain more resources,

4. If they had to discuss their work with an assessor, and,

5. If they were planning safety improvements.

They were also asked which diagram was easier to understand and which would be easier to update. The interviews were transcribed and analysed thematically.

## Results

### Data Analysis

The interviews were transcribed and the interview questions were used as a framework for the analysis. Responses were analysed to identify what safety concerns the participants mentioned and how many concerns they mentioned. In the second part of the procedure when they were asked to circle their concerns on the diagram the responses were collated from the diagram. Finally, questionnaire responses were analysed by counting the number of participants who chose each response.

### Initial perceptions of quality and safety without using a process map

When participants were asked about safety and quality problems in the CBAC service without referring to a diagram, six participants cited between two and six issues each. One participant did not identify any safety or quality problems. The patient safety concerns were categorized as either administrative, clinical or co-ordination across healthcare boundaries and are shown in Table 1. The most frequent concerns (9) noted were associated with the clinical work. Safety risks arising from the difficulty of co-ordinating patient care across the boundaries of the healthcare system were also commonly noted (6).

**Table 1:** Quality and safety concerns elicited during interviews

Area of concern identified		Number of participants	CBAC service function	
Limitations of software - *e.g. *diary management, INR values allowed, acknowledgement of team communications		4	Clinical	

Availability of backup from doctor or specialist for difficult cases		3	Clinical	

Gaining information from other healthcare providers such as GP, Primary Care Trust or laboratory		3	Co-ordination across healthcare boundary	

Follow-up of patients who do not attend an appointment		2	Administration	

Availability of information following discharge from hospital ward		2	Co-ordination across healthcare boundary	

Lack of training to identify additional health problems in patients		1	Clinical	

Patients' understanding of medication instructions		1	Clinical	

Suitability of patients referred to the service		1	Co-ordination across healthcare boundary	

### Perceptions of quality and safety when using the process maps

All participants stated that they understood both the sequential flow diagram and the HTA and no participant asked further questions about the format of the diagrams. Two pharmacists noted a missing step (reflecting a difference particular to one clinic location) and indicated on both diagrams where the step should have been represented. All other participants reported that the diagrams accurately reflected the system as they knew it.

In order to compare participants' responses to the diagrams, the quality and safety problems circled on the two diagrams were categorized as administrative, referring to the process of enlisting patients, or clinical work, referring to patient monitoring. There were no concerns about co-ordination across clinical boundaries. Table 2 summarises which areas of the process were identified as problematic by participants for each process map. More safety and quality problems were identified in relation to clinical work than administrative work. Only clinical processes containing safety problems were highlighted on the sequential flow diagram whereas both administrative and clinical processes were highlighted on the HTA diagram. More safety problems were identified using the HTA, than with the sequential flow diagram.

**Table 2:** Quality and safety problems identified on sequential flow diagram and hierarchical task analysis

Diagram	Administrative task step (sequential) or goal (HTA)			Clinical task step (sequential) or goal (HTA)				Total
	**Obtain** **patient** **list**	**Invite** **patient**	**Schedule** **patient**	**Obtain** **medical** **history**	**Obtain** **INR**	**Determine** **dose &** **duration**	**Conclude** **appointment**	

Sequential flow diagram	0	0	0	0	3	4	1	8

Hierarchical task analysis	2	1	1	3	1	2	1	11

We also examined within participant responses. Table 3 shows the areas each participant circled on each diagram. Participants were generally not consistent in circling the same areas of the process on both maps. Only one participant (participant 5) circled the same part of the process on both diagrams. The participants' identification of risk also varied between the open-ended questions asked prior to seeing the diagrams and those indicated during the "hands-on" work with the diagrams. For example, the participant who did not mention any concerns when asked without the diagrams did indicate two concerns on the HTA.

**Table 3:** Quality and safety problems identified by each participant

Participant	Administrative task step (sequential) or goal (HTA)			Clinical task step (sequential) or goal (HTA)			
	**Obtain** **patient** **list**	**Invite** **patient**	**Schedule** **patient**	**Obtain** **medical** **history**	**Obtain** **INR**	**Determine** **dose &** **duration**	**Conclude** **appointment**

1				H	S	S	S

2			H	H		S	

3	H					H	H

4				H	S	H	

5					H, S	S	

6						S	

7	H	H					

H = participant identified potential safety problem on HTA

S = participant identified potential safety problem on sequential flow diagram

Where both H and S are indicated, the order reflects the order in which the participant viewed the diagrams

The results of the questionnaire are summarized in Table 4. The HTA was clearly preferred for discussing a problem with a colleague, but the sequential flow diagram was preferable for detailing other problems. The HTA was perceived as being easier to develop to a further level of detail.

**Table 4:** Questionnaire results

Question	Flow diagram	HTA	No Preference	No answer
1. Which diagram was easiest to understand?	3	2	2	0

2. Which would you choose to explain your work to someone who works outside the system?	3	3	1	0

3. Which would you choose to discuss a problem with a colleague who does the same job?	1	5	1	0

4. Which would you choose to explain your work to management so that you can gain support and resources?	3	3	1	0

5. Which would you choose to discuss an issue with an assessor?	1	3	2	1

6. Thinking about the *safety *problems discussed, which is the most useful for detailing the analysis/improvements to be done?	1	2	4	0

7. Thinking about the *other problems *we discussed, which is the most useful for detailing the analysis/improvements to be done?	4	0	1	2

8. If you had time and resources, which would be easier to develop to further level of detail (granularity)?	1	5	1	0

Total	17	23	13	3

## Discussion

This study showed that healthcare practitioners' perceptions about the risks in their system were different depending on whether they were reflecting on the risks without a process map, working with a sequential flow diagram or working with a hierarchical diagram (HTA). The results suggest that the type of representation chosen for use in quality improvement work is important because improvement efforts will be influenced by how the process is represented: variation in the safety problems that practitioners identified was related to the type of process map they used.

The results of this study suggest that improvement efforts might need to be based on more than one type of representation to ensure that all aspects of the process are captured. Using both sequential and hierarchical diagrams might yield a more comprehensive view of the process than using one alone. This is in agreement with Marsolek and Friesdorf [9] who advocated the use of both sequential and hierarchical diagrams for improving work systems and processes.

What a representation captures is clearly important. In the present study, co-ordination across organizational boundaries was not represented in the process maps. Both diagrams were focused on tasks performed by the healthcare professionals within the bounded context of the clinic, but risks often emerge just outside those boundaries in the patient's behaviour and in the liminal zone between different care services. Communication and liaison across organizations and the movement of patients between services were cited as some of the biggest problems when participants were asked open ended questions without the diagrams, and this has been confirmed by ongoing safety improvement work within the CBAC, including root cause analyses. Some of these issues were captured in a limited way by the HTA through its high-level goals, but not by the sequential flow diagram.

During the mapping process our reflective notes showed that two central questions must be decided early when constructing a process map: defining the system boundary and the granularity required. We decided to define our system by the entry and exit of a patient under care of the CBAC service. However, as noted, problems were perceived and experienced at the boundaries of the system. To ensure that the important aspects of the process are captured, the construction of a process map should therefore proceed iteratively with emerging information about where the risks in the system are located. Preliminary investigation into risks identified through incident reporting and interviewing clinicians will be necessary to determine where the boundary of the process map should be set, and this might change as the mapping process continues. If the focus of the improvement work is to be the interface between different parts of the healthcare system (in our case, with GPs, the Primary Care Trust and hospitals), mapping should involve participants with knowledge of these services.

The other key decision is the granularity of the diagram. The HTA method includes a stopping rule for formalizing how much detail is represented [16]. There is no similar guidance for flow diagrams, with the possibility that some parts of the process might be shown in more detail than others, thus biasing subsequent improvement work. A further practical difficulty is how to represent the maps as their size increases quickly. An electronic representation can, with the appropriate software, be easier to revise, particularly with a hierarchical map. Berlingieri et al. [18], for example, offer a web-based view of a process map in which sections expand or contract to ease visualisation.

Which of these methods healthcare professionals will choose to use will probably be a pragmatic choice based on the time available and how easy each method is to use. Reflecting on the experience of constructing the maps we found that the two diagrams each had advantages and disadvantages. The HTA was highly structured and thus easier to produce graphically and easier to revise as the mapping progressed. It offered flexibility in representing important goals which did not correspond to specific acts at specific times but which represented ongoing issues that could be triggered at any time, such as seeking help from a peer, patient education, or tasks that were purely cognitive. On the other hand, the timing of some parts of the work was much easier for us to handle within the flow diagram than the HTA. The flow diagram was harder to adapt because additional details have to be added within the process steps, creating branches and loops. The information gleaned from standard operating procedures was easier to represent with the HTA, but information gained from interviews and observations was easier to represent with the flow diagram.

Despite HTA being little used in healthcare compared to flow diagrams and anecdotal reports that it is hard for healthcare professionals to understand, we found it was as readily accepted as the flow diagram. The HTA was preferred by participants for discussing their work with a colleague, suggesting that the representation of goals in the HTA is important in providing context and enabling people with similar levels of expertise to improve the system. Healthcare work is driven by the need to achieve the goals of patient care despite variability in the patients, the demands on the service and the support available, both technological and social. The representation of those goals in the hierarchical structure of the HTA is therefore important, especially if communicating with others who understand those goals. The unpredictability of healthcare and the professional autonomy of healthcare practitioners are also better encompassed by the HTA with its focus on the goal to be achieved rather than the precise method used.

This study was exploratory and has suggested that the form of a process map does influence perceptions of the quality and safety problems in a service and different maps draw attention to or away from different aspects of the work. Interviewing clinicians to elicit concerns not readily captured by process maps was also found to be important, and should be regarded as an essential preliminary step to determine the boundary of the process map and to identify known risks. However, the study was limited by a number of factors. First, the size of the sample was constrained by the small number of people in the service with sufficiently detailed knowledge to participate. A difficulty of conducting such studies is that each clinical micro system is unique and is likely to contain only small numbers of experts who can contribute. Second, we asked participants to simply identify their quality and safety concerns, whereas most quality improvement work will follow a structured method to identify where improvements can be made. For example, HFMEA proceeds by asking specific questions about how a process could fail at each step [4]. The study did not evaluate whether different types of process maps are more useful for particular quality improvement methods, but this is an important question to investigate in subsequent work. Third, in order to compare the two diagrams the content had to be standardised, which meant that the maps were constructed by the researchers. The practitioners were then presented with a finished map to work with (although they were asked to review and update it). In most quality improvement work the team-based mapping process is an important part of the process of generating shared understanding about how work is carried out in reality and where risks are located [6]. We acknowledge that in this study this did not occur and this might have affected the results.

Fourth, a related issue is that the study design necessitated standardising the diagrams so that they each contained the same content, but it is possible that the diagrams are most effective at representing different parts of the process. This could be examined in further work. Finally, the clinical setting examined in this study involved tasks that were relatively well structured and well defined. This work could be extended by examining different clinical systems with different clinical demands and different professional groups. We recognise, in particular, that the differing autonomy of different healthcare professions in different roles has implications for whether a flow diagram or HTA should be preferred.

## Conclusions

The results of this study indicated that the layout of a process map can influence healthcare practitioners' perceptions of quality and safety problems in a process. It is therefore important to carefully consider the most suitable type of process map to use and whether to use more than one representation in order to capture different aspects of the clinical work and ensure that all relevant aspects are shown. Although the process map is often seen as a preliminary step in undertaking quality improvement work, it is a vitally important aspect of how that work proceeds.

## Competing interests

The authors declare that they have no competing interests.

## Authors' contributions

LC conceived of the study, participated in its design, collected the data, constructed the process maps, and drafted the first version of the manuscript. JA and HP participated in the design of the study and the analysis of the data and revised subsequent versions of the manuscript. JB participated in the design of the study and reviewed the manuscript. All authors read and approved the final manuscript.

## Pre-publication history

The pre-publication history for this paper can be accessed here:

http://www.biomedcentral.com/1472-6963/10/7/prepub
